# Liposomal Elongation Factor-1α Triggers Effector CD4 and CD8 T Cells for Induction of Long-Lasting Protective Immunity against Visceral Leishmaniasis

**DOI:** 10.3389/fimmu.2018.00018

**Published:** 2018-01-30

**Authors:** Abdus Sabur, Sudipta Bhowmick, Rudra Chhajer, Sarfaraz Ahmad Ejazi, Nicky Didwania, Mohammad Asad, Anirban Bhattacharyya, Utsa Sinha, Nahid Ali

**Affiliations:** ^1^Infectious Diseases and Immunology Division, CSIR-Indian Institute of Chemical Biology, Kolkata, India

**Keywords:** visceral leishmaniasis, cationic liposome, vaccine, elongation factor-1α, Th1/Th2 response

## Abstract

Despite advances, identification and formulation of safe and effective vaccine for long-lasting protection against leishmaniasis is still inadequate. In this study, we have identified a novel antigen, leishmanial elongation factor-1α (EF1-α), as an immunodominant component of solubilized leishmanial membrane antigens that reacts with visceral leishmaniasis (VL) sera and induces cellular proliferative and cytokine response in PBMCs of cured VL subjects. Leishmanial EF1-α is a 50 kDa antigen that plays a crucial role in pathogen survival by regulating oxidative burst in the host phagocytes. Previously, immunodominant truncated forms of EF1-α from different species of *Leishmania* have been reported. Formulation of the *L. donovani* 36 kDa truncated as well as the cloned recombinant EF1-α in cationic liposomes induce strong resistance to parasitic burden in liver and spleen of BALB/c mice through induction of DTH and a IL-10 and TGF-β suppressed mixed Th1/Th2 cytokine responses. Multiparametric analysis of splenocytes for generation of antigen-specific IFN-γ, IL2, and TNF-α producing lymphocytes indicate that cationic liposome facilitates expansion of both CD4^+^ as well as CD8^+^ memory and effector T cells. Liposomal EF1-α is a novel and potent vaccine formulation against VL that imparts long-term protective responses. Moreover, the flexibility of this formulation opens up the scope to combine additional adjuvants and epitope selected antigens for use in other disease forms also.

## Introduction

The most serious form of leishmaniasis is caused by visceralizing parasites belonging to *Leishmania donovani* complex. The disease is characterized by severe manifestations, such as hepatosplenomegaly, fever, pancytopenia, hypergammaglobulinemia, immune suppression, and death without appropriate treatment (1). Antileishmanial chemotherapies are long, expensive, and have adverse side effects. This compounded with the emergence of drug-resistant strains and increased HIV co-infection in developing countries underscore the need for an effective and safe vaccine (2).

Cure from leishmaniasis in most cases leads to attainment of long-term immunity to reinfection raising the prospect of developing a preventive vaccine (3). Despite several advances including three licensed vaccines (Leishmune^®^, Leishtec^®^, and Canileish^®^) against canine visceral leishmaniasis (VL), a preventive vaccine for human use in the near future is still elusive (4–9). The major obstacles in vaccine development are to characterize the immunology of long-lasting protective immunity and to devise safe and effective strategies to achieve this goal.

Chronically progressive VL is marked by subverted host antigen-presenting cells (APCs) and consequent T cell dysfunction (10). Restoration of Th1 type immune response and phagocyte activation ensures cure. Induction of similar recall response is, therefore, considered essential for resistance to subsequent infection (11, 12). However, apart from generating protective response, the major challenge in vaccine development is to maintain the responses for a long duration, preferably lifelong.

Considering safety parameters, defined antigen-based vaccines are preferred, and consequently a majority of vaccines against human leishmaniasis under clinical investigation but yet to get licensed are protein based, notably, stable emulsion of MPLA/GLA with recombinant fusion antigens LEISH-F1, LEISH-F2, and LEISH-F3 (8).

Although elicitation of cell-mediated responses is considered to be involved in antileishmanial adaptive immunity, most protein-based vaccines fail to induce cellular immune response without adjuvant or delivery systems. Adjuvants and/or delivery systems can be devised to prevent extracellular degradation and premature clearance of antigens. Previous preclinical and clinical studies suggest antigen persistence as crucial aspect for generation of long-lasting immunity against leishmaniasis. In addition to dose sparing, without compromising antigen persistence, adjuvant-delivery system may facilitate uptake and presentation for induction of cellular immune responses (13). Liposomes facilitate antigen uptake and presentation into the MHC class II pathway of phagocytic APCs, including macrophages and DCs. Positively charged liposomes are known to be preferentially taken up by APCs compared to their neutral and anionic counterparts (14). Moreover, cationic liposomes facilitate cross-presentation to MHC class I pathway, and hence, are efficient inducers of CD8^+^ T cell response (15).

A major thrust in vaccine development has been on rational identification and design of defined antigens. However, it is unlikely that a single recombinant antigen can induce uniform and strong immune response in heterogeneous population with diverse HLA haplotypes. Moreover, devising defined multiantigenic or multiepitope (cocktail or fusion) vaccine formulations require identification of potent component antigens or epitopes. The key to identification of these protective antigens is to determine the role played by the antigens in pathogenicity of VL and to determine the intrinsic epitope induced immunogenicity in human context. A number of potent antigens, such as KMP11, triose phosphate isomerase, A2, NH36, H2B, CPA, CPB, etc., have been screened for their immunogenicity with human PBMCs, so as to rationally overcome the limitations of animal studies (16–18).

This prompted us to use redundant method to identify an immunogenic antigen from solubilized leishmanial membrane antigens (SLA), a mixture of dominant polypeptides enriched from total leishmanial membrane antigens (LAg). SLA formulated with cationic liposomes conferred almost complete protection as a prophylactic as well as therapeutic vaccine against *L. donovani* challenge infection (19, 20). In the present study, we purified a polypeptide of 36 kDa from SLA in its native form and identified it as a truncated form of elongation factor-1α (EF1-α) of *L. donovani*, using matrix-assisted laser desorption ionization–time of flight (MALDI-TOF/TOF) mass spectrometry. Nandan et al. were the first to identify *L. donovani* EF1-α as an important virulence factor that shares a unique biochemical feature in binding and activation of tyrosinephosphatase-1 (SHP-1) to downregulate inducible nitric oxide synthase (iNOS) expression within host phagocytes (21). The antigen was also biochemically and structurally characterized and compared with mammalian homologue of EF1-α to identify distinct structural and biochemical differences that may lead to potential drug targets (22). Herein, we investigated the immunogenicity of 36 kDa antigen from SLA that reacts with anti-EF1-α antibodies, in inducing proliferation, IFN-γ, and IL-12 from PBMC of cured VL patients. We account the protective efficacy of both the anti-EF1-α reactive 36 kDa antigen and recombinant EF1-α in cationic liposomes against *L. donovani* infection and explored the immune responses correlating with long-lasting protection in murine model. We also investigated the role of cationic liposomes for induction of effector CD4^+^ and CD8^+^ T cells and memory responses for long-lasting immunity against VL with entrapped protein antigen.

## Materials and Methods

### Parasites and Culture Condition

Promastigotes of *L. donovani*, strain AG83 (ATCC^®^ PRA413™) maintained by passage in Syrian hamsters were cultured at 22°C in medium 199 (pH 7.4) with 10% heat-inactivated fetal bovine serum, 2 mM l-glutamine, 100 U of penicillin G sodium, and 100 µg of streptomycin sulfate per milliliter and sub-cultured in the same medium at an average density of 2 × 10^6^ cells/ml (23).

### Preparation of SLA

SLA from *L. donovani* promastigotes, was prepared as detailed earlier (24). The detergent octyl β d-glucopyranoside used for solubization was removed from SLA preparation by serial dialysis in 20 mM PBS (pH 7.2) and SLA was stored at −20°C until use. The amount of protein obtained from 1.0 g cell pellet, as assayed by the method of Lowry et al. (25), was approximately 2 mg.

### Gel-Filtration Chromatography

SLA was fractionated by gel-filtration chromatography on a Sepharose CL-6B column (Sigma-Aldrich) of 80 cm height and 1 cm radius. Briefly the column was equilibrated with 5 mM Tris–HCl (pH 7.6) with 0.01% octyl-β-d-glucopyranoside buffer. 2 ml of SLA (0.75 mg/ml) in 5 mM Tris–HCl with 0.01% octyl-β-d-glucopyranoside was applied to the column and flow through was maintained at 2 ml per minute. Individual fractions of 1 ml were collected and assessed for proteins by measuring the absorbance at 280 nm. The column was calibrated with molecular mass standards (Bio-Rad) thyroglobulin (610 kDa), aldolase (158 kDa), ovalbumin (44 kDa), and myoglobin (17 kDa). Fractions containing protein were run on SDS-PAGE. The 36 kDa protein-enriched fractions were lyophilized, dialyzed, and stored at −20°C until use.

### Cloning, Expression, and Purification of *L. donovani* EF1-α

PCR amplification of the gene corresponding to EF1-α was carried out using standard conditions similar to that described by Saiki et al. (26). The ORF of *L. donovani* (AG83) (GenBank assembly accession: GCA_001989955.1; NCBI, BioProject Accession—PRJNA297706) EF1-α was amplified using forward 5′-ACCATGGGCAAGGATAAGGTG-3′ and reverse 5′-CTTCTTCGCAGCCTTCG-3′ primers. The amplified gene was cloned into the multiple cloning site of plasmid pET15b. The cloned vector was transformed into *Escherichia coli* Rosetta for expression by isopropyl-β-d-thiogalactoside (IPTG) induction. After lysis of IPTG-induced bacterial cells by sonication and centrifugation, the pellets of inclusion bodies were resuspended in 50 mM Tris–HCl (pH 8) buffer containing 200 mM NaCl and 8 M urea and 10 mM imidazole and then centrifuged to collect the supernatant. The supernatants were allowed to bind to Ni-NTA agarose matrix equilibrated in the same buffer for 2 h at 4°C. After binding, the matrix was washed thrice with 50 mM Tris–HCl, 200 mM NaCl, and 8 M urea buffer containing 50 mM imidazole and 0.1% TritonX-100 to remove non-specifically bound endotoxins, followed by another three washes without the detergent. The proteins were eluted by 500 mM imidazole in 50 mM Tris–HCl, 200 mM NaCl, and 8 M urea buffer and serially dialyzed with decreasing urea concentration in PBS buffer (pH 7.2). The residual endotoxins were removed by passing through a 10 kDa filtration cut (Millipore). The endotoxin level was measured by chromogenic quantitation method using Limulus Amebocyte Lysate QCL-1000^TM^ kit (Lonza) according to manufacturer’s instructions and found to be 0.6 ± 0.1 EU/ml. The purity of rEF1-α were confirmed by SDS-PAGE (27) and protein concentration was determined by Lowry’s method (25). Confirmation of the expressed proteins was done by immunoblotting using anti-EF1-α antibody (Upstate Biotechnology, CA, USA).

### Human Subjects

The human subjects of the present study consisted of 10 VL patients, admitted to the School of Tropical Medicine (Kolkata, India), mainly from Bihar and West Bengal. Each of the 10 patients were sampled for blood before and after successful treatment with amphotericin B by i.v. drip in dextrose solution on alternate days of total dosage of 20 mg/kg of body weight. Ten individuals from CSIR-Indian Institute of Chemical Biology (IICB) (Kolkata, India) were included as healthy controls (HCs) with no previous history of VL and 10 individuals with disease other than leishmaniasis were used for control experiments. The study was approved by the institutional Ethical Committee on Human Subjects, CSIR-IICB and also by School of Tropical Medicine (Kolkata, India) (Ref. No. CREC-STM/319). Written informed consents were obtained from all patients and donors for blood sampling. Plasma and PBMCs were obtained from the heparinized blood samples of treated VL patients and HCs by density sedimentation (Histopaque-1077; Sigma-Aldrich) (28).

### Immunoblot Analysis

Protein samples were separated by SDS-10% PAGE according to the method of Laemmli (27). Following electrophoretic transfer of the resolved proteins to nitrocellulose membrane, the membrane strips were blocked for 2 h with 5% BSA in 100 mM Tris-buffered saline, pH 7.6, washed once with 0.05% Tween-20 in TBS (washing buffer), and incubated for 1 h with individual human sera or monoclonal anti-EF1-α antibody (Upstate Biotechnology) in 1:1,000 or 1:100 dilutions, respectively, in washing buffer. The blots were then washed three times and incubated again for 1 h with 1:1,000 dilution of HRP-conjugated goat anti-human IgG or goat anti-mouse IgG antibodies (Bangalore Genei), followed by five washes as before except the last wash, which was done without Tween-20. Enzymatic activity was revealed with luminol (MERK) to detect chemiluminescence.

### Lymphoproliferation and Cytokine Assay

PBMCs were isolated from heparinized blood by density sedimentation (Histopaque-1077; Sigma-Aldrich) as described previously (28). The PBMCs (1 × 10^6^ cells/200 μl/well) were cultured in triplicate in a 96-well flat-bottom plate (Nunc) and stimulated with SLA (10 µg/ml) or EF1-α (2.5 µg/ml) for 72 h at 37°C at 5% CO_2_. After 72 h, supernatants were analyzed for IFN-γ and IL-12 by ELISA (BD Pharmingen), according to the manufacturer’s instructions. The remaining cells were pulsed with 1 μCi of [^3^H]-Thymidine (Amersham Biosciences) per well and incubated for another 16–18 h and harvested on glass fiber paper. Thymidine incorporation was measured after incubation for 16–18 h in a β-scintillation counter (Beckman Instruments) (24).

### Liposomal Formulation and Entrapment of Antigens

Liposomes were prepared as detailed earlier by dispersion of lipid film of 1,2-distearoyl-*sn*-glycero-3-phosphocholine, cholesterol, and stearylamine at a molar ratio of 7:2:2 in either 1 ml of 0.02 M PBS (pH 7.4) alone or containing 0.5 mg/ml of purified native or recombinant EF1-α (23). The mixture was then vortexed and sonicated in an ultrasonicator (Misonix, New York, NY, USA) for 40 s, followed by incubation at 4°C for 2 h. The excess free antigens were removed by ultracentrifugation at 100,000 × *g* for 1 h at 4°C. The proteins entrapped in liposome were estimated indirectly by deducting the amount washed proteins from the initial using Lowry’s method (29).

### Immunization of Mice

BALB/c mice were bred in the animal facility of CSIR-IICB (Kolkata, India). At the onset of experiments, all the mice were 4–6 weeks old. The studies were performed according to the institutional guidelines of the Committee for the Purpose of Control and Supervision on Experimental Animals, Ministry of Environment and Forest, Govt. of India, and approved by the Institutional Animal Ethics Committee (147/1999/CPSCEA) of CSIR-IICB.

For post immunization and short-term protective efficacy studies, a set of 40 BALB/c mice in four groups were immunized by intraperitoneal injections of 200 µl of PBS (G1), empty cationic liposome (G2), 2.5 µg of 36 kDa purified EF1-α in PBS (G3) or incorporated in cationic liposome (G4) in a total volume of 200 µl. Mice were boosted two times at 2-week intervals. Ten days after the last booster, serum (blood) samples were collected, and five animals from each group were sacrificed to collect spleens for study of immune response post immunization, and the remaining five mice per group were challenged with virulent parasites. For studies with recombinant antigens, a similar experimental set of 40 mice in four groups PBS (R1), Liposome (R2), recombinant EF1-α (R3), and liposomal rEF1-α (R4) were used and the experimental set up was same as discussed for native antigen. For long-term protective studies, 20 mice equally distributed in four groups PBS control (F1), empty cationic liposome (F2), 36 kDa purified EF1-α (F3), and liposomal rEF1-α (F4) were immunized similarly and were challenge infected 12 weeks post last booster dose. In T cell subset depletion experiments, 35 mice were distributed in seven groups- PBS (D1), liposome (D2), liposomal EF1-α (D3), liposomal EF1-α + anti-CD4 (D4), liposomal EF1-α + anti-CD8 (D5), liposomal EF1-α + anti-CD4 + anti-CD8 (D6), liposomal EF1-α + control IgG (D7). The D4 mice received 1 mg of monoclonal antibodies GK1.5 for depletion of CD4^+^ T cells and D5 mice received monoclonal antibodies GK 2.43 for depletion of CD8^+^ T cells, while D6 mice received both, GK1.5 and 2.43, 2 days before each vaccination and 2 days after last vaccination. This schedule resulted in greater than 95% depletion of CD4^+^ and CD8^+^ T cells, as assessed by FACS analysis. For D7 mice rat IgG was used as a control in depletion experiments. The mice were infected 10 days after the final booster dose. All the experimental sets were repeated two times.

### Infectious Challenge and Evaluation of Infection

Ten days and twelve weeks after the last booster the mice were intravenously challenged with 2.5 × 10^7^ freshly transformed stationary-phase promastigotes in 200 µl PBS as described earlier (23, 30). After 90 days, the mice were sacrificed to determine the parasite burden in liver and spleen (31). The amastigote nuclei were counted by the microscopic examination of Giemsa-stained impression smears of liver and spleen. The parasite load was expressed as Leishman Donovan units calculated as number of amastigote nuclei per 1,000 cell nuclei multiplied by organ weight in milligrams (Stauber). Viable *L. donovani* parasites in the mice were enumerated by limiting dilution assay (LDA) as previously reported (29). Tissues from liver and spleen were weighed and homogenized in complete Schnieder’s insect media with 10% FBS. The homogenized tissue were cultured in fivefold serial dilutions at 22°C for 21 days in 96-well tissue culture plates (Nunc) with 1 mg tissue homogenate at the initial well. The culture plates were examined for the presence of transformed motile promastigotes every 7 days for 21 days. The reciprocal of the highest dilution at which viable promastigotes were detected was considered to be the concentration of parasites per mg of tissue. The total organ parasite burdens were calculated by multiplying the reciprocal per mg dilution value with the total weight of the respective organs in milligrams.

### Determination of DTH, Antibody, Cell Proliferation, and Cytokine Responses

DTH response was evaluated post immunization and infection by measuring the difference in footpad swelling 24 h following intradermal injection of test footpad with 40 µg of LAg in 50 µl PBS with that of control PBS-injected footpad using a constant-pressure caliper (Starrett Company) (23). Sera from both immunized and infected mice were analyzed by ELISA for the presence of antigen-specific IgG1 and IgG2a antibodies as described previously (23). Similar ELISA method was used to determine reactivity of human sera with SLA and EF1-α. For cell proliferation and cytokine assay, single cell suspensions of splenocytes were prepared in RPMI 1640 complete medium containing 10% FBS, 100 U/ml penicillin G sodium, 100 µg/ml streptomycin sulfate, and 50 µM β-mercaptoethanol (Sigma-Aldrich) (complete medium). Then, the cells were cultured in triplicate at a density of 2 × 10^5^ cells/200 μl/well in 96-well flat-bottom plate (Nunc) and stimulated with antigens (2.5 µg/ml) in the presence or absence of anti-CD4 and anti-CD8 monoclonal antibody (1 µg/10^6^ cells; BD Pharmingen). The cells were incubated for 72 h at 37°C in a humified chamber containing 5% CO_2_. After 72 h incubation, culture supernatants were collected, and the concentrations of IFN-γ, IL-4, IL-12p40, TNF-α, IL-10 (BD Pharmingen) and TGF-β (R&D Systems) were quantified by ELISA in accordance with the manufacturers instructions. For TGF-β, supernatants were acidified prior to the assay (32). The remaining cells were pulsed with 1μCi of [^3^H]-Thymidine (Amersham Biosciences) per well and incubated for another 16–18 h and harvested on glass fiber paper. Thymidine uptake was measured in a β-scintillation counter (Beckman Instruments) (24).

### Multiparametric Flow Cytometry

Splenocytes from immunized animals were plated at 1 × 10^6^ cells/well in 24-well flat-bottom plate and stimulated with 5 µg of rEF1-α for 12 h at 37°C. Brefeldin A (10 µg/ml) was added to the cultures 2 h before harvesting. The cells were washed in PBS containing 0.1% NaN_3_/1% FCS and stained with PE-conjugated anti-CD3 and either PerCP5.5-conjugated anti-CD4 or APC-Cy7-CD8 mAb (BD Pharmingen) for 30 min at 4°C. The cells were then permeabilized with 1× BD Perm2 reagent for 10 min at 4°C, followed by washing with the above-described buffer containing 0.1% saponin and the cells were fixed using cytoperm kit. Then the intracellular cytokines were stained with APC-conjugated anti-IFN-γ, FITC-conjugated anti-IL2 and PE-Cy7-conjugated anti-TNF-α for 30 min at 4°C. After proper washing, cells were analyzed on BD LSR-Fortessa flow cytometer on at least 100,000 events and analyses were carried out by FlowJo software as described previously (33). For memory markers, the splenocytes apart from CD3-PE, CD4-PE-Cy7, and CD8-APC-Cy7, surface markers were also stained with APC-conjugated anti-CD62L and PerCP5.5-conjugated anti-CD44. After fixation the cells were analyzed similarly as described above.

### Stastitical Analysis

GraphPad Prism 5.0 software was used for all the statistical analysis. For groups involving human sera and PBMCs, two-tailed, Mann–Whitney *U* test was used for comparision between two groups. ROC curves for ELISA were plotted to determine the cutoff values at 95% confidence intervals to discriminate between VL patients and HCs. For experimental mice studies, the differences between the data sets were analyzed by ANOVA with Bonferroni’s post test corrections. For comparisons between two groups, unpaired two-tailed *t* tests were performed. A value of *p* ≤ 0.05 was considered significant.

## Results

### Immunological Recognition of 36 kDa Leishmanial Antigen with VL Patient Sera, and Anti-EF1-α Monoclonal Antibody

**Figure 1:**
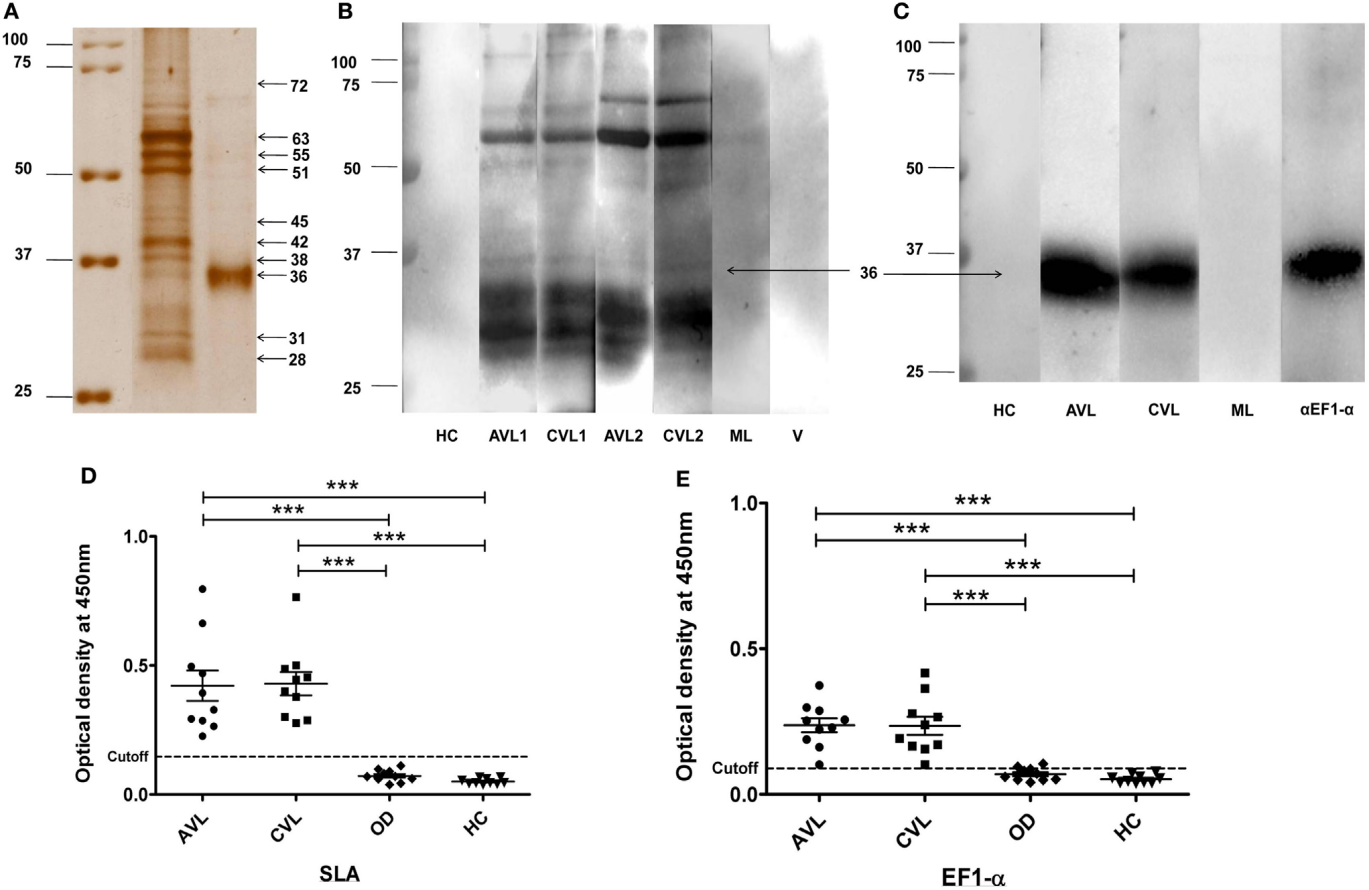
36 kDa purified fraction of solubilized leishmanial membrane antigens (SLA) recognized by visceral leishmaniasis (VL) sera and anti-elongation factor-1α (EF1-α) monoclonal antibody. Silver stained SDS-PAGE of SLA and 36 kDa purified antigen **(A)**. SLA (5 µg) and purified 36 kDa antigen (3 µg) protein were separated on 10% SDS-PAGE. Marker lane was merged to indicate the molecular weight of each band (raw images in Figure S1 in Supplementary Material). **(B)**. 4 µg of SLA was electro-transferred from SDS-PAGE gel to nitrocellulose membrane. Blots were probed with sera from two VL patients before (AVL1, AVL2) and after treatment (CVL1, CVL2), one healthy (HC), one malaria (ML), and one viral fever (V) control subjects. **(C)**. Western blot analysis of 36 kDa antigen purified from SLA probed with sera from VL patient, before (AVL1) and after (CVL1) treatment, healthy control (HC), ML and by anti-EF1-α monoclonal antibodies (αEF1-α) (Upstate, CA). **(D,E)** ELISA of SLA and 36 kDa purified antigen of SLA with sera of VL subjects before (AVL) and after cure (CVL) along with healthy (HC) and other disease controls (OD). Each sample was run in duplicates. ROC curve was plotted with 95% confidence interval between the AVL and HC groups.

Solubilized leishmanial membrane antigens is a solubilized pool of membrane antigens obtained from *L. donovani* promastigotes. SDS-PAGE of SLA reveals a low complexity composition consisting of approximately eight to ten major bands varying from 72–25 kDa (Figure 1A). The search for antigens as potent vaccine candidates from SLA was done by western blot analysis using sera from two individual VL patients, before and after treatment (Figure 1B). The blots were also probed with sera from healthy, malaria (ML) and viral disease controls which did not show specific reactivity to SLA (Figure 1B). 36 kDa band from SLA along with other bands showed reactivity with sera from active VL patients with persistent recognition after successful chemotherapy in both the patients. The 36 kDa antigen in its native form was purified using Sepharose CL-6B gel-filtration chromatography. To identify the 36 kDa band, fraction enriched with 36 kDa protein was separated by SDS-PAGE (Figure 1A; Figure S1 in Supplementary Material). The mass spectrometry analysis of tryptic digest of 36 kDa band identified the protein as EF1-α of *L. donovani* (accession no. AAL08019) with eight peptides matched and 18% sequence coverage (Table S1 in Supplementary Material). Immunoblotting and ELISA of 36 kDa antigen was performed to verify the recognition of this antigen by sera from VL patient before and after treatment (Figures 1C,E). Sera from VL patients, before and after treatment reacted specifically while sera from healthy and ML controls did not react to the 36 kDa purified antigen from SLA (Figures 1C,E). Reactivity of the 36 kDa antigen with anti- EF1-α monoclonal antibody confirmed the identity of the protein as truncated form of EF1-α. ELISA of individual VL sera before and after treatment were carried out with control, healthy and other diseases. The cutoff values of reactivity were determined to be 0.1465 and 0.0895 for SLA and 36 kDa antigen, respectively (Figures 1D,E).

### Induction of Th1-Type Cellular Responses to EF1-α in VL Patients at Cure

**Figure 2:**
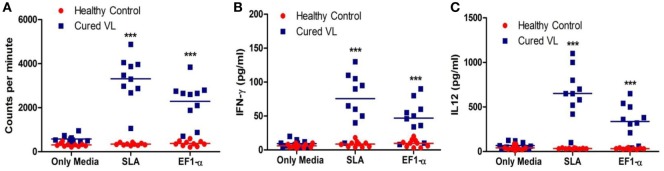
Antigen-specific lymphroliferative and cytokine response in PBMCs of cured visceral leishmaniasis (VL) patients. PBMC were isolated from healthy controls (HCs) (*n* = 10) represented as red dots, VL patients after treatment (*n* = 10) represented as blue square blocks. PBMC were stimulated with medium, elongation factor-1α (EF1-α) (2.5 µg/ml), solubilized leishmanial membrane antigens (SLA) (10 µg/ml) for 96 h. **(A)** Lymphoproliferation were measured by [^3^H] thymidine incorporation after another 16–18 h of culture and is expressed in counts per minute. **(B)** After 96 h, IFN-γ were measured by ELISA from the supernatants of cultures. **(C)** IL-12 from the 96-h culture supernatants were measured. Each symbol represents the proliferation or cytokine production for one patient. Thick horizontal lines represent the mean values of each group and ***p < 0.001 vs. HC.

As 36 kDa truncated EF1-α demonstrated reactivity with VL patients’ sera, ability of this antigen to stimulate PBMCs isolated from posttreated VL patients for *in vitro* proliferative and cytokine responses were assayed. At cure, PBMCs from 9 out of the 10 patients studied elicited lymphoproliferative responses (Figure 2A) and also secreted higher levels of IFN-γ and IL-12 in comparison to PBMCs from healthy individuals (Figures 2B,C) when stimulated with SLA. PBMCs from eight cured patients showed significantly higher levels of proliferation, IFN-γ and IL-12 production (*p* < 0.05) compared to HCs when stimulated with EF1-α.

### Liposomal EF1-α Mediated Induction of Mixed Th1/Th2 Immune Responses in BALB/c Mice

**Figure 3:**
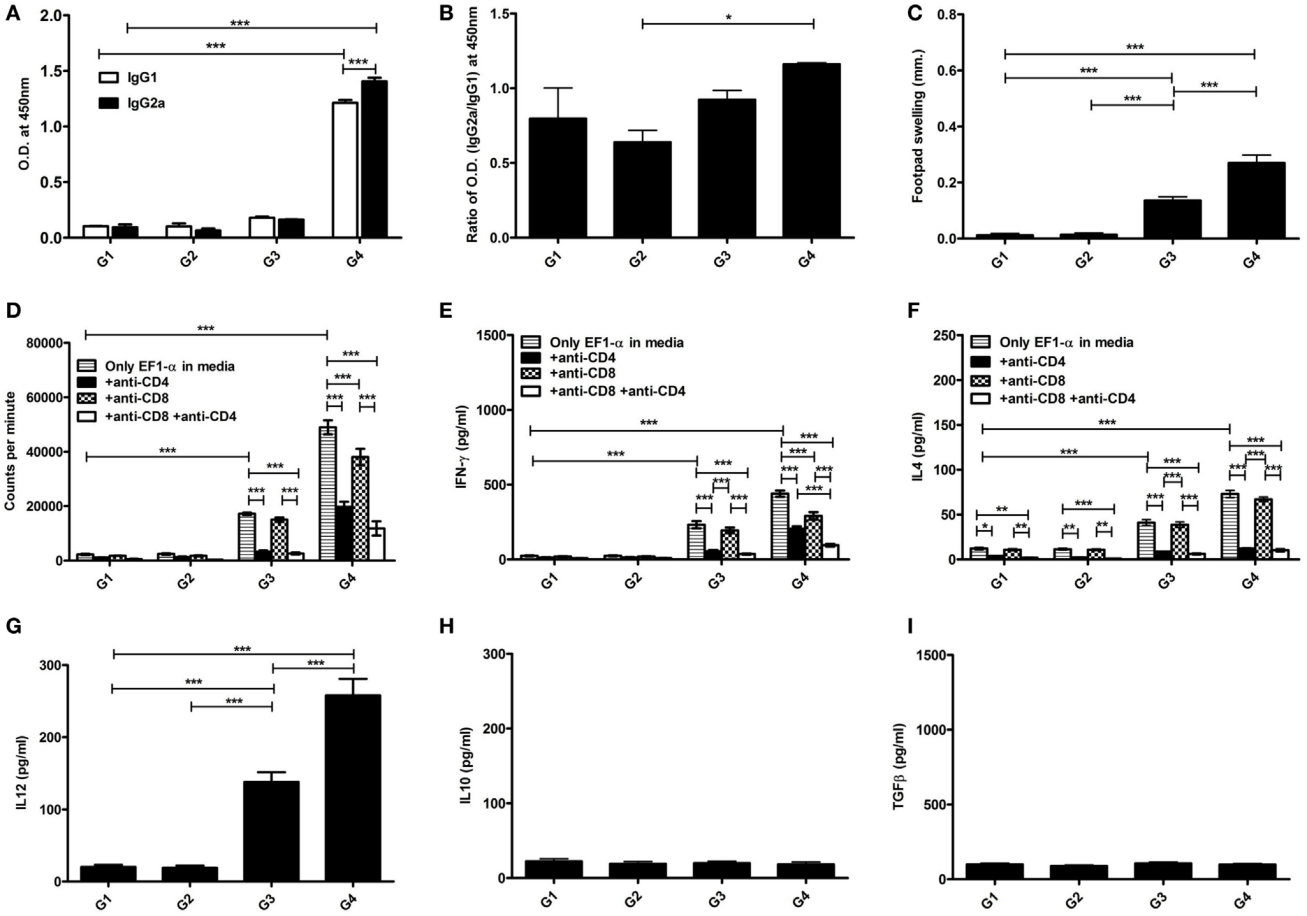
Antigen-specific immune responses in BALB/c mice post immunization. Prechallenge antibody, DTH, proliferative and cytokine responses in mice immunized with PBS (G1), liposome (G2), purified 36 kDa elongation factor-1α (EF1-α) in PBS (G3) and entrapped in cationic liposome (G4) were evaluated. **(A)** Ten days after the last immunization, serum samples were collected and assayed for antigen-specific IgG1 and IgG2a antibodies at 200 dilutions by ELISA. **(B)** The ratio of absorbance between IgG2a and IgG1 were plotted. **(C)** DTH responses were measured as the difference (in millimeters) between the thickness of the test (LAg-injected) and control (PBS-injected) footpads at 24 h. Spleens were collected and splenocytes were stimulated *in vitro* with EF1-α (2.5 µg/ml) for 72 h in presence or absence of anti-CD4 and anti-CD8 monoclonal antibody(1 µg/10^6^ cells). **(D)** Antigen-specific splenocyte proliferation was determined by [^3^H] thymidine incorporation after another 16–18 h of culture and expressed as counts per minute. Each sample was examined in triplicates from five mice per group. The culture supernatants were also assayed for IFN-γ **(E)**, IL-4 **(F)**, IL-12 **(G)**, IL-10 **(H)**, TGF-β **(I)** by capture ELISA. Data are representative of mean ± SE of five individual mice per group examined in duplicates (*p < 0.05; **p < 0.01; ***p < 0.001).

**Figure 4:**
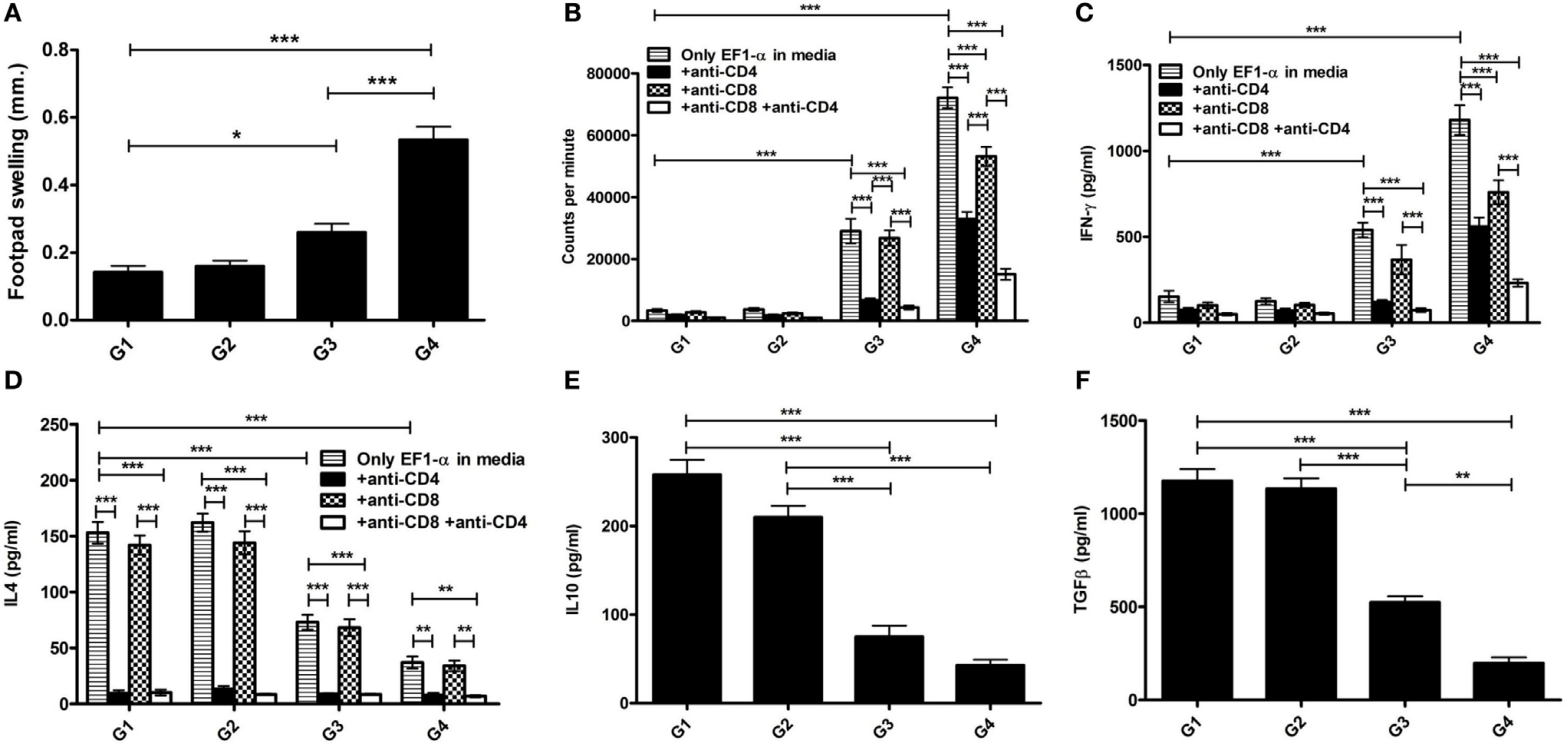
Antigen-specific immune responses in immunized BALB/c mice 3 months post challenge infection with virulent *Leishmania donovani*. Three months after challenge, infection in immunized mice groups PBS (G1), cationic liposome (G2), purified 36 kDa elongation factor-1α (EF1-α) (G3) liposomal 36 kDa EF1-α (G4), DTH, proliferative and cytokine responses were evaluated. **(A)** DTH responses were measured as the difference (in millimeters) between the thickness of the test (LAg-injected) and control (PBS-injected) footpads at 24 h. Spleens were collected and splenocytes were stimulated *in vitro* with EF1-α (2.5 µg/ml) for 72 h in presence or absence of anti-CD4 and anti-CD8 monoclonal antibody(1 µg/10^6^ cells). **(B)** Antigen-specific splenocyte proliferation was determined by [^3^H] thymidine incorporation after another 16–18 h of culture and expressed as counts per minute. Each sample from five mice per group was examined in triplicate. The culture supernatants were also assayed for IFN-γ **(C)**, IL-4 **(D)**, IL-10 **(E)**, and TGF-β **(F)** by capture ELISA. Data are representative of mean ± SE of five individual mice per group each examined in duplicates (*p < 0.05; **p < 0.01; ***p < 0.001).

The antibody and recall cell-mediated immune (CMI) responses were evaluated 10 days after the last vaccination. Liposomal EF1-α vaccination resulted in higher titers of antigen-specific both IgG2a and IgG1 (Figures 3A,B). Mice that received free EF1-α had very low level of both the isotypes. The ratio of IgG2a:IgG1 in mice immunized with liposomal EF1-α was >1 (Figure 3A). *In vivo* DTH response was estimated by measuring antigen induced footpad swelling. Mice receiving EF1-α either in PBS or entrapped in cationic displayed a significantly higher DTH response as compared to control PBS and empty liposomal groups (Figure 3C) indicating induction of antigen-specific CMI responses. Splenocytes isolated from the mice that received liposomal EF1-α immunization exhibited stronger proliferative response (Figure 3D) and stimulation of both IFN-γ and IL-4 than the control PBS immunized mice (Figures 3E,F) (*p* < 0.001). Blocking experiments with anti-CD4 and anti-CD8 monoclonal antibody to assess the relative contributions of CD4^+^ and CD8^+^ T cells revealed that both the treatments blocked the proliferation and IFN-γ release, whereas addition of anti-CD4 inhibited the IL-4 production in liposomal EF1-α immunized mice. Again, levels of other type 1 cytokines IL-12 and TNF-α were measured in the splenocytes supernatants of liposomal EF1-α-vaccinated mice. A higher level of IL-12 (Figure 3G) (*p* < 0.001) was observed. Importantly, no significant changes in the level IL-10 and TGF-β production were observed (Figures 3H,I). In order to determine whether the immune response triggered post immunization could resist parasite infectivity, the mice were evaluated for DTH, proliferation, and cytokine response, 90 days after infection with virulent *L. donovani* parasites. A similar surge in DTH response (Figure 4A), antigen-specific proliferation of splenocytes (Figure 4B) and IFN-γ response (Figure 4C) were observed in liposomal EF1-α-vaccinated mice. Interestingly, while a surge in the levels of IL-4, IL-10, and TGF-β were observed in control mice, the regulatory cytokines (Figures 4D–F) were significantly inhibited in liposomal EF1-α immunized group. In addition to higher IFN-γ and IL-12 levels, secretion of TNF-α was also enhanced in liposomal EF1-α immunized mice (data not shown).

### Short- and Long-term Protective Efficacies of Liposomal EF1-α and the Involvement of Both CD4^+^ and CD8^+^ T Cells

**Figure 5:**
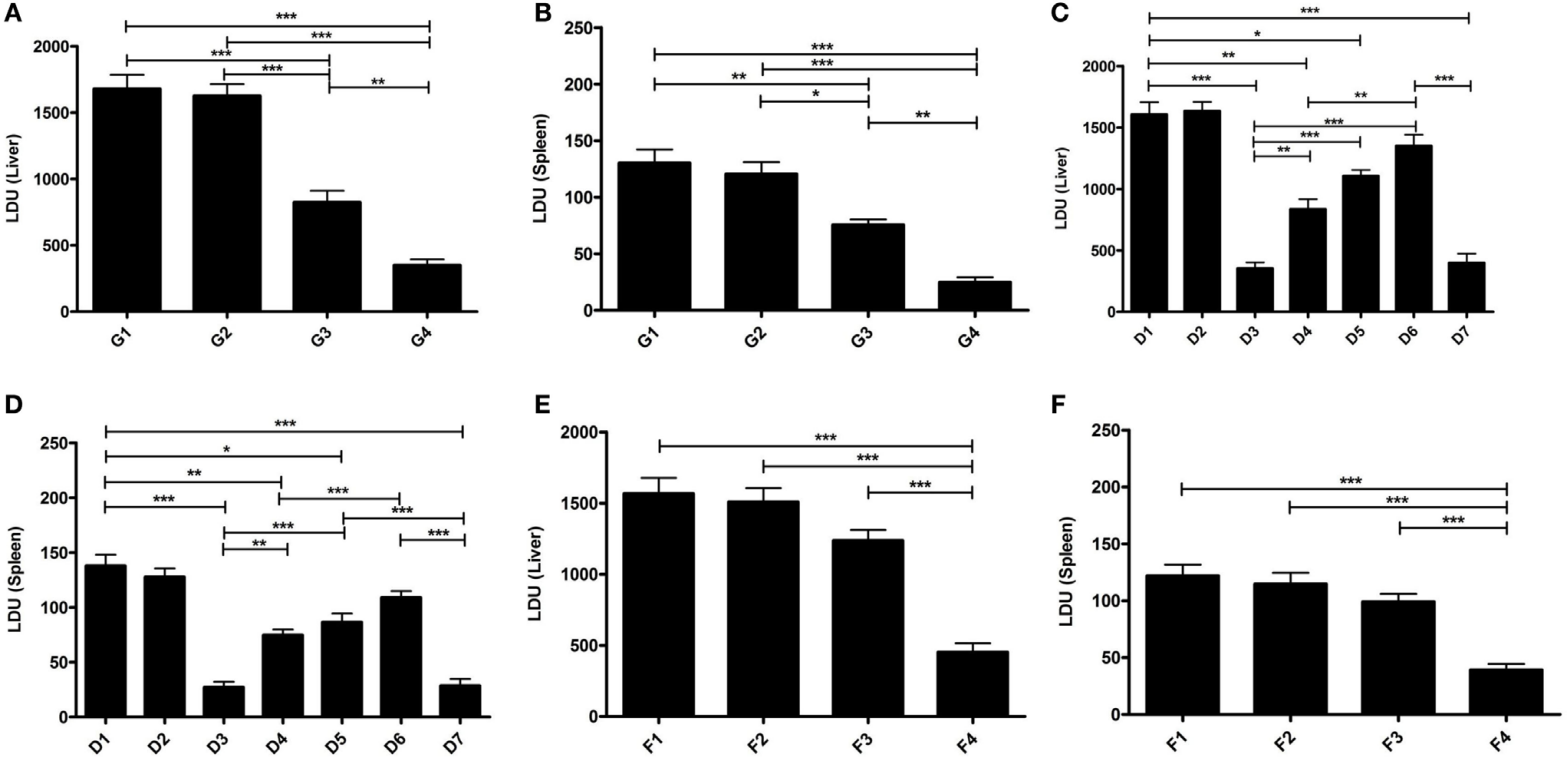
Protective efficacy (short and long) of elongation factor-1α (EF1-α) alone or entrapped in cationic liposomes in normal and CD4 and/or CD8 depleted BALB/c mice. Liver **(A)** and spleen **(B)** parasite burdens in mice challenged 10 days after final immunization with PBS (G1), cationic liposome (G2), purified 36 kDa EF1-α (G3), and liposomal 36 kDa EF1-α (G4) as Leishman Donovan Units. Parasite burdens in cationic liposomal EF1-α-vaccinated mice after depletion of CD4^+^ or/and CD8^+^. Mice were vaccinated intraperitoneally three times with either PBS (D1), liposome (D2), liposomal EF1-α (D3), liposomal EF1-α + anti-CD4 (D4), liposomal EF1-α + anti-CD8 (D5), liposomal EF1-α + anti-CD4 + anti-CD8 (D6), liposomal EF1-α + control rat IgG (D7) and challenge infected after 10 days of final immunization. Depletion with either anti-CD4 or anti-CD8 monoclonal antibody, or both, 2 days before each vaccination and 2 days after last vaccination. Liver **(C)** and spleen **(D)** parasite burdens were measured 90 days after challenge as Leishman Donovan Units. Liver **(E)** and spleen **(F)** parasite burdens of mice challenge infected 12 weeks after final immunization with PBS (F1), cationic liposome (F2), purified 36 kDa EF1-α (F3), and liposomal 36 kDa EF1-α (F4) were measured 90 days after challenge as Leishman Donovan Units. All the results are represented as mean ± SE of five individual mice per group (*p < 0.05; **p < 0.01; ***p < 0.001).

Our data demonstrated that mice immunized with EF1-α alone, were resistant to hepatic infection at partial level at 90 days after challenge infection (Figure 5A). Again, mice receiving liposomal EF1-α acquired higher resistance (79%), significantly higher than that afforded by the free antigen (*p* < 0.01). In BALB/c mice persistence of *L. donovani* in the spleen causes concomitant development of considerable organ-specific pathology similar to that seen in the human kala-azar. It was, therefore, more important to evaluate the impact of vaccination in this organ. In spleen, immunization with liposomal EF1-α demonstrated 81% protection (Figure 5B). The reduction in parasitic load was statistically higher than that by EF1-α (*p* < 0.01), which by itself could induce partial reduction in spleen parasite burden. In order to confirm the relative contribution of both CD4^+^ and CD8^+^ T cells to vaccine-elicited protection in liposomal EF1-α mice, both the T-cell subset depletion experiments were performed. As shown in Figures 5C,D, liposomal EF1-α mice effectively control the parasite replication in both liver and spleen after 90 days of challenge infection. In contrast, treatment of liposomal EF1-α immunized mice with anti-CD4 and anti-CD8 antibodies partially reversed parasite multiplication in both liver and spleen, which was not observed when the mice were treated with control antibody. Moreover, the immunized mice depleted of both CD4^+^ and CD8^+^ T cells failed to control infection and showed higher parasite burden, confirming liposomal EF1-α-protectively immunized mice were incapable of controlling challenge infection when both CD4^+^ and CD8^+^ T cells were depleted. Finally, in order to evaluate the durability of the immunity induced by the liposomal EF1-α vaccine, another set of animals were immunized similarly but challenged 12 weeks after boosting. Ninety days after infection when parasite loads were well expressed in both liver and spleen, mice were sacrificed and parasite loads were quantified. Mice vaccinated with liposomal EF1-α were protected against parasite growth in both liver (71%) and spleen (68%) (Figures 5E,F). On the contrary, free EF1-α immunization failed to provide any durable protection against visceral infection. These data indicate that this liposomal formulation of EF1-α protein vaccine was able in sustaining long-term immunity in mice.

### Induction of Protective Immunity by Recombinant EF1-α in Cationic Liposomal Formulation

**Figure 6:**
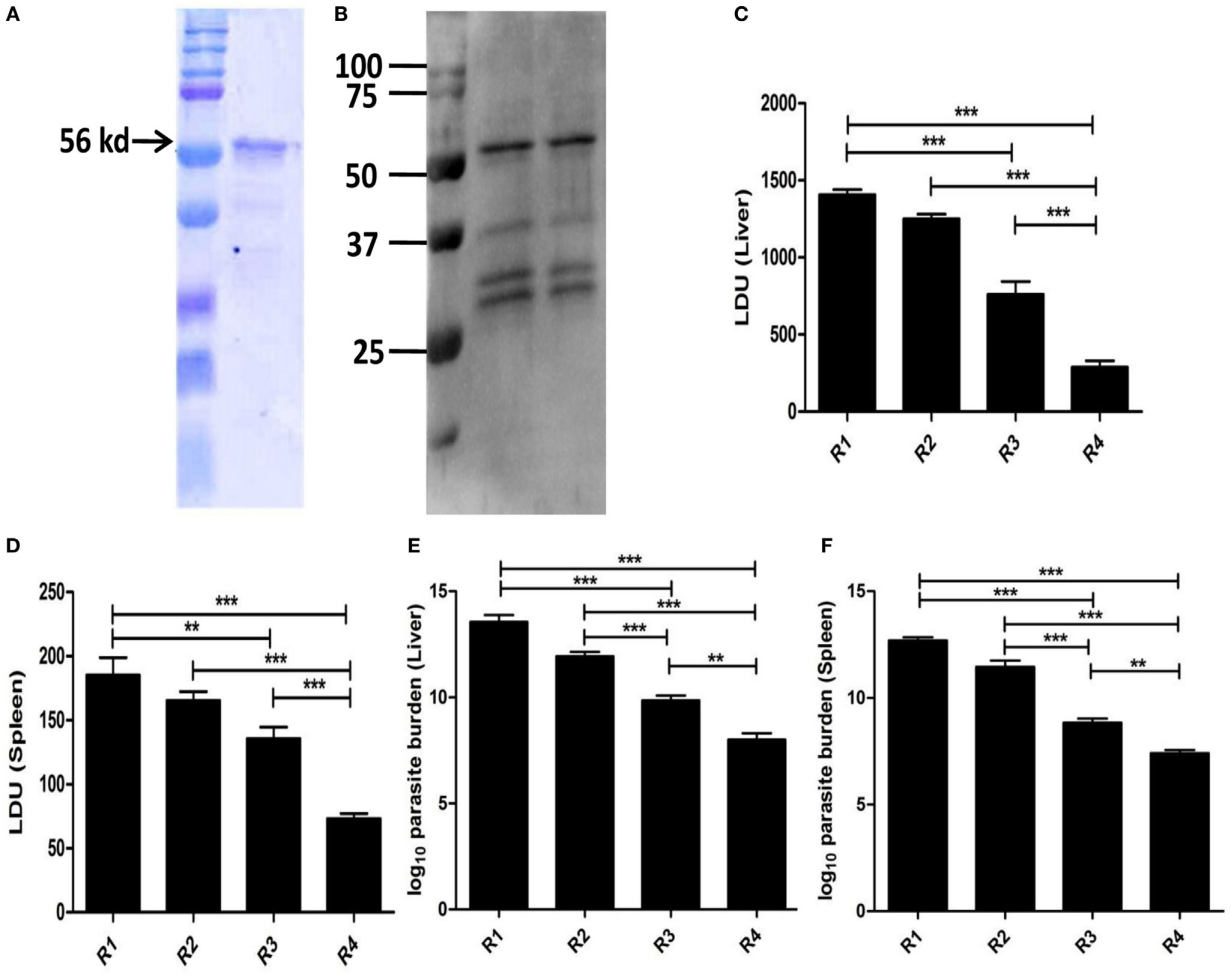
Protective efficacy of recombinant elongation factor-1α (EF1-α) expressed and isolated from *Escherichia coli*. The gene from *Leishmania donovani* EF1-α was cloned into the multiple cloning site of plasmid pET15b, transformed, and overexpressed in Rossetta strain of *E. coli* by isopropyl-β-d-thiogalactoside induction. The recombinant antigen was purified from overexpressed bacterial lysate under denaturing condition by Ni-NTA chromatography, residual endotoxins were removed by 10-kd cut (Millipore) and purity was confirmed by SDS-PAGE **(A)**. The antigen was then electro-transferred to nitrocellulose membrane. The identity of the purified antigen was verified by probing with anti-EF1-α monoclonal antibody (Upstate, CA, USA) **(B)**. Mice were vaccinated intraperitoneally three times with 2.5 µg of rEF1-α alone (R3) or entrapped in cationic liposomes (R4) at 2-week intervals. Control groups received PBS (R1) or empty liposomes (R2). Ten days after the last immunization, the mice were challenged intravenously with 2.5 × 10^7^ promastigotes of *L. donovani*. Liver **(C)** and spleen **(D)** parasite burdens were measured 90 days after challenge as Leishman Donovan Units, and estimates of viable parasites in liver **(E)** and spleen **(F)** were determined by Limiting Dilution assay. The results are mean ± SE of five individual mice per group (*p < 0.05; **p < 0.01; ***p < 0.001).

The whole gene from *L. donovani* EF1-α was cloned with overhang codons of six histidine amino acids and overexpressed in Rossetta strain of *E. coli* by IPTG induction. The recombinant antigen was purified and the purity >95% was confirmed by SDS-PAGE (Figure 6A). The antigen was then electro-transferred to nitrocellulose membrane. The identity of the purified antigen was verified by probing with anti-EF1-α monoclonal antibody (Upstate, CA, USA) (Figure 6B). In order to compare whether the recombinant whole EF1-α could impart similar immune response and protection as native isolated rEF1-α, similar vaccination and infection protocol were applied and the immune response following immunization and infection were determined. The immune response with liposomal rEF1-α in both post immunization and post infection studies were concominantly similar to the native rEF1-α (Figures S4 and S5 in Supplementary Material). Furthermore, our data demonstrated that mice immunized with rEF1-α alone were resistant to hepatic infection at partial level at 90 days after challenge infection (Figure 6C). Again, mice receiving liposomal rEF1-α acquired higher resistance (83%), significantly higher than that afforded by the free antigen (*p* < 0.01). In spleen, immunization with liposomal rEF1-α demonstrated 71% protection (Figure 6D). The reduction in splenic parasitic load in rEF1-α immunized mice was statistically higher than that of controls (*p* < 0.01). Estimation of viable parasites by LDA indicates a 7- to 6-log fold reduction in parasite burden in liver (Figure 6E) and in spleen (Figure 6F), respectively.

### Multifunctional T Cell and Memory Response to Liposomal rEF1-α in BALB/c Mice

**Figure 7:**
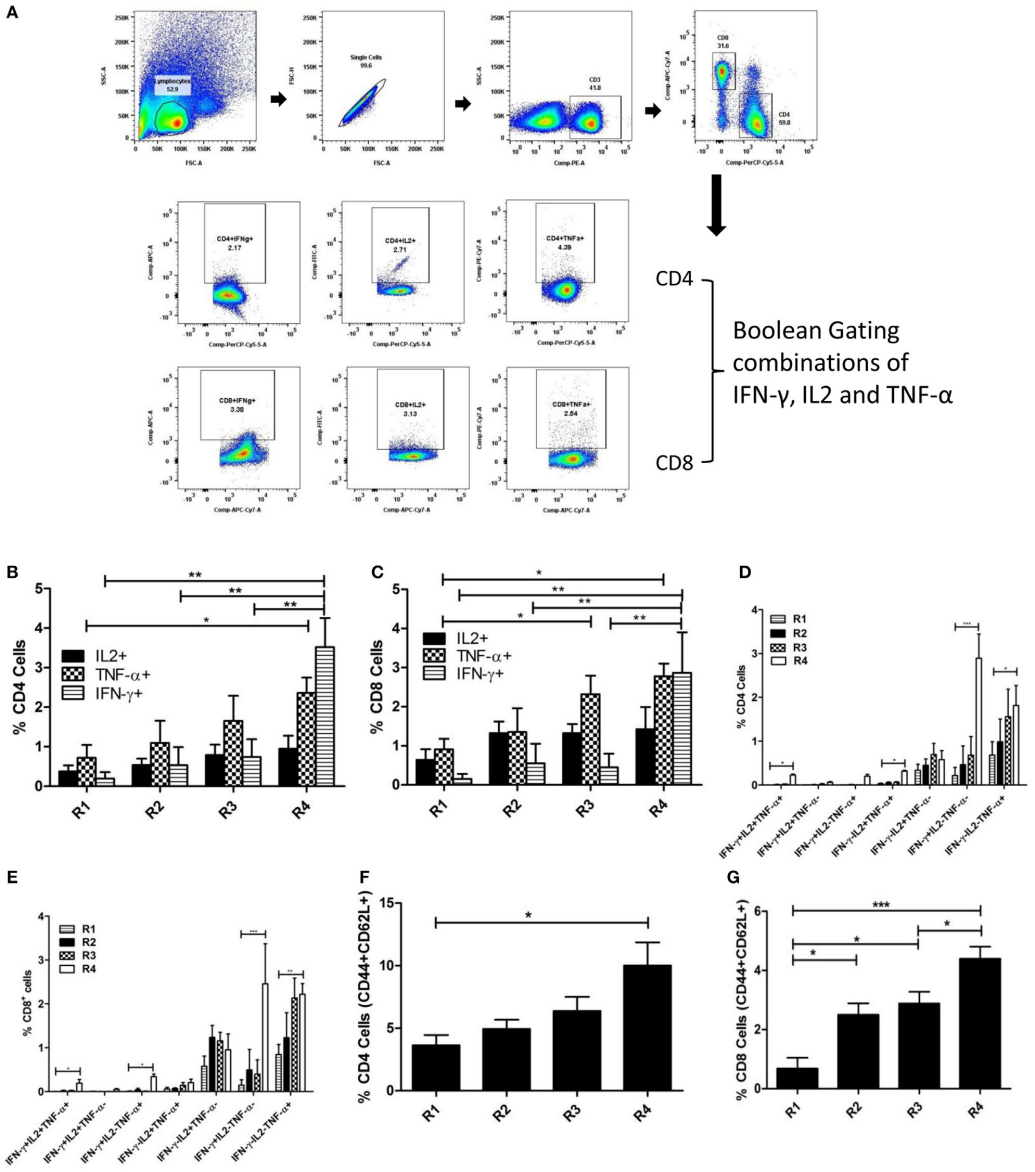
Multifunctional T cell response to liposomal rEF1-α in BALB/c mice. Mice were immunized intraperitoneally three times with 2.5 µg of rEF1-α alone (R3) or entrapped in cationic liposomes (R4) at 2-week intervals (*n* = 3 per group). Control groups received PBS (R1) or empty liposomes (R2). Ten days after the last immunization, spleens were isolated and splenocytes (10^6^ cells/ml) were stimulated *in vitro* with rEF1-α (2.5 µg/ml) for 12 h. Splenocytes were then stained for various surface markers and internally trapped cytokines. Multiparametric analysis of cytokines and memory markers were performed using Boolean gating Strategy by using FloJo analysis software. **(A)** Representative plot of gating strategy of cytokine producing T cells. **(B)** Plot of percentage CD4^+^ cells producing IFN-γ, IL2, TNF-α individually and **(D)** frequency of CD4^+^ cells expressing the three cytokines in seven possible combinations. **(C)** Plot of percentage CD8^+^ cells producing IFN-γ, IL2, TNF-α individually and **(E)** frequency of CD8^+^ cells expressing the three cytokines in seven possible combinations. Plots of frequency of CD4^+^ and CD8^+^ cells double positive for CD44 and CD62L has been represented in **(F,G)**, respectively. Results are expressed as means ± SEM of three independent experiments *p < 0.05; **p < 0.01; ***p < 0.001.

In order to determine the functional involvement of different subsets of T cells in imparting protective immunity against murine VL, splenocytes of immunized mice were stimulated with and stained for various surface markers and internally trapped cytokines. Both the percentage of CD4^+^ and CD8^+^ T cells producing IFN-γ and TNF-α were significantly higher in the liposomal rEF1-α group (Figures 7B,C). The multiparametric analysis of cytokines using Boolean gating Strategy (Figure 7A; Figure S2 in Supplementary Material) indicates that although the proportion of double and triple cytokines producing T cells are lower than single cytokine producing T cells, liposomal rEF1-α-vaccinated mice show enhanced expansion of IFN-γ and/or TNF-α as well as multifunctional triple positive T cells as compared to control PBS and liposomal group (Figures 7D,E). Moreover, there was a significant increase in frequency of both CD4^+^ and CD8^+^ T cells which are double positive for effector memory markers CD44 and CD62L (Figures 7F,G).

## Discussion

Development of an effective vaccine has been an essential aim for the control of leishmaniasis. Major impediment to develop safe subunit vaccine strategy against this disease lies in identifying relevant antigens and devising formulations that can impart sustained protective immunity (16). Herein, we have identified a novel leishmanial antigen, *L. donovani* EF1-α and evaluated the vaccine potential of the antigen both in its native truncated form as well as in recombinant form against VL. We report EF1-α in a liposomal formulation induces long-term protective immunity against *L. donovani* infection in BALB/c mice by activation and differentiation of polyfunctional T cells.

Immunological screening is a rational approach to identify antigens that can potentially trigger immune responses for impairment of pathogenicity (12). Sand fly salivary antigens, known for their important role in immunomodulation and parasite infectivity, have been exploited as transmission blocking vaccine candidates against leishmaniasis (34, 35). Moreover, several antigens from the parasite have been reported to induce immunomodulatory effect that can be exploited for designing vaccine against leishmaniasis. Rationally, a number of parasite antigens including sand fly salivary components have been identified as potent vaccine candidates based on preliminary reactivity with sera from infected dogs and humans (36–38). Nevertheless, persisting reactivity to VL sera at cure, where immune response skews toward protective type, is more suitably exploited to identify putative protective antigens. Our results demonstrate consistent serological reactivity of 36 kDa SLA antigen in VL patients both before and after treatment. Previously, we have reported SLA to induce excellent protective as well as therapeutic immunity against *L. donovani* infection in BALB/c mice (19, 20). As a reservoir of protective subunit antigens, we reported the protective efficacy of different components of SLA (30). Previously uncharacterized immunodominant 36 kDa antigen of SLA, was purified in its native form and identified as EF1-α, a translation factor belonging to GTPase superfamily, of *L. donovani* by MALDI-TOF/TOF. The reactivity of the purified fraction of anti-EF1-α monoclonal antibody and sera of active and cure VL patients further confirms its identity. Genomic analysis, however, reveals EF1-α from *L. donovani* as an ~50 kDa protein, encoded by a stretch of 1,347 nucleotide gene sequence (gene sequence id.—FR799604.2). Although different truncated forms of EF1-α have been reported, its mechanism and evolutionary function have not been characterized. For instance, Piedro-Quintello et al. identified a 29 kDa immunodominant *L. mexicana* antigen as a different molecular form of EF1-α (39). Moreover, in a separate study, different immunodominant fractions ranging from 30 to 36 kDa were identified as truncated forms of *L. infantum* EF1-α (40). Leishmanial EF1-α shares a unique biochemical feature in binding and activation of tyrosinephosphatase-1 (SHP-1) to downregulate iNOS expression inside host phagocyte (21). The attenuation of iNOS-mediated oxidative burst is crucial for survival of *Leishmania* in the host cells. This important pathogenicity associated protein, despite being reported to dominantly react to VL sera, has not been tested for its immunogenicity and vaccine potential thereof (40).

A CMI response is essential for clearance of intracellular pathogen. Therefore, apart from proteome serology, identification and prediction of protective antigen has been rationally based upon the ability to stimulate CMI response in PBMCs of healed or asymptomatic VL subjects. A number of prospective vaccine antigens, like HASPB,- KMP11, A2, EF2, TSH, STM, CPB, CPC, NH36, etc., were identified by their ability to induce lymphoproliferative and Th1 cytokine response in PBMCs of protected subjects (41–44). Herein, we demonstrate a comparative analysis of recall response induced by purified 36 kDa truncated EF1-α and SLA. While PBMCs of 9 out of 10 cured VL patients proliferated in response to SLA as compared to 8 in case of native EF1-α, similar upsurge in secretion of IFN-γ and IL-12 were also observed. The ability of SLA to induce better recall response can be due to heterogeneity of antigens suggestive of better efficacy of multiantigenic/epitope vaccines as compared to subunit antigens. To our knowledge, this is the first report demonstrating stimulation of Th1 response by cured VL patients in response to EF1-α, making it a strong constituent candidate for defined multiantigenic vaccine strategy. Serological and cellular immunoreactivity of the leishmanial virulence-associated protein EF1-α prompted further evaluation for its immunogenicity and protective efficacy in *Leishmania* model.

*Leishmania* survives and establishes chronic pathogenesis inside host phagocytes mainly by evading and attenuating the microbicidal effector functions (45). Priming of immune response to activate and augment oxidative killing of amastigotes by the host phagocytes is one of the major objectives of vaccine design. Evaluation of immune response triggered post immunization with both native and recombinant EF1-α formulated with cationic liposome disclose induction of cellular immune response as evident from strong DTH response as compared to PBS and liposome controls. Moreover, an upsurge in antigen-stimulated production of IFN-γ, IL-12, and TNF-α in the vaccinated splenocytes was observed. The Th1 cytokine IFN-γ along with supplementary effect of TNF-α activate JAK-STAT signaling pathway to upregulate iNOS production and hence promote oxidative burst (46). IFN-γ is also known to promote antibody class switching to IgG2a type (47). Concomitant to cytokine secretion a dominant titer of IgG2a over IgG1 in Liposomal EF1-α-vaccinated mice was observed. However, along with IFN-γ the increased level of IL-4 in the vaccinated mice indicate that a mixed Th1/Th2 response were generated post immunization. In order to understand whether the immune response triggered by EF1-α and its liposomal formulation was sufficient to resist *L. donovani* infection, parasite burden and the consequent immune response post infection were studied.

Unlike other infection models of *Leishmania* where infection onset is early and leads to self healing response especially in C5BL6 mice, infection with *L. donovani* AG83 is chronically progressive in BALB/c mice. Infectious challenge of *L. donovani* AG83 in BALB/c mice leads to establishment of hepatic and splenic infection at 8 weeks and peaking up to 16 weeks post challenge and hence parasite load in liver and spleen were studied at 90 days post challenge to determine vaccine efficacy (31, 48). *Leishmania* infection modulates the host immune response to secrete immunosuppressive cytokines, such as IL-10 and TGF-β, which favors parasite survival by deactivating the host phagocytes (10). Neutralizing effect of immunosuppressive cytokines to the effector function of Th1 cytokines have been reported in different forms of leishmaniasis (49). Our results suggest that the increased levels of IL-4, IL-10, and TGF-β in the control infected mice is concomitant to the higher parasite burden observed in both liver and spleen. In contrast to the control groups, a significant fall in the levels of Th2 and regulatory cytokines, with high Th1 cytokines in the vaccinated mice is correlated with the resistance to infection following challenge with virulent parasites. Therefore, apart from maintaining high DTH and Th1 cytokine responses, downregulation of immunosuppressive cytokines is crucial for parasite clearance. The response and protective immunity generated from crude 36 kDa antigen identified as truncated EF1-α was furthermore validated with whole recombinant EF1-α that imparts similar protective responses. A considerable decrease in parasite load was observed in liver and spleen of mice challenged with virulent parasites 10 days (short-term study) and 90 days (long-term study) after last immunization with liposomal EF1-α. Interestingly mice immunized with EF1-α alone could partially resist infection in short-term studies but failed to impart long-term protection.

Experimental as well as clinical studies with first generation vaccines suggest that protein-based antigens without suitable adjuvant and delivery system fail to impart durable protection, a requisite feature for a successful vaccine against VL (50, 51). Evidence to long-lasting protection can be extracted from studies with live vaccines and infection induced acquired immunity imparting durable resistance to reinfection (51). A persisting antigen or pathogen is crucial for the expansion of multifunction CD4^+^ and CD8^+^ T cells that can boost effector as well as memory response (52–54). Our results with *ex vivo* depletion studies demonstrate that cationic liposome formulation of EF1-α facilitates activation of IFN-γ producing CD4^+^ as well as CD8^+^ T cells. Moreover, *in vivo* depletion of either CD4^+^ or CD8^+^ T cells at the time of vaccination partially reversed the inhibition of parasite multiplication. The reversion of protection was observed when both the T cell subsets were depleted, suggesting both CD4^+^ and CD8^+^ T cells are activated by vaccination with liposomal EF1-α. Direct evidence of the involvement of effector CD4^+^ and CD8^+^ T cell was confirmed with recombinant EF1-α by multiparametric flow cytometric analysis. Previously, we have reported cationic liposomes as an efficient delivery system for entry of antigens into the MHC class I pathway and thus are efficient inducers of CD8^+^ T cell responses (15). Herein, we investigated cationic liposome-mediated expansion of single cytokine producing as well as multifunctional effector CD4^+^ and CD8^+^ T cell by multiparametric flowcytometric analysis. Various studies have shown that induction of single cell polyfunctionality of T cells leads to better vaccine-induced protection against leishmaniasis (55, 56). Our results demonstrate that immunization with liposomal EF1-α leads to induction of significantly higher population of antigen-specific triple positive (IFN-γ^+^TNF-α^+^IL2^+^) CD4^+^ and CD8^+^ T cells in comparison to infected untreated controls. However, the proportion of single and double cytokine producing (IFN-γ^+^ and/or TNF-α^+^) CD4^+^ as well as CD8^+^ T cells was more than triple-positive multifunctional T cells. Thus, both multifunctional and single or double cytokine producing effector CD4^+^ and CD8^+^ T cells are generated in liposomal EF1-α-vaccinated mice. Apart from cytokine secreting T cells, our results demonstrate that vaccination with liposomal EF1-α leads to enhanced differentiation of CD44 and CD62L positive T cells. The CD44 and CD62L positive central memory T cells are crucial for generation of long-lasting vaccine-mediated immunity (57). The findings are consistent to our previous reports on liposomal vaccine-mediated durable protection against VL (58). We report cationic liposome-mediated induction of multifunctional effector as well as memory T cells.

Liposomal EF1-α is a novel Th1-stimulating vaccine formulation that imparts significant protection in BALB/c mice against challenge with *L. donovani* after a short as well as long-term immunization protocol. Our results also indicate that induction of Th1 response with elevated levels of IFN-γ, IL-12, and TNF-α and an inhibition of IL-4, IL-10, and TGF-β levels can tilt the immune system toward protection against VL. Furthermore, studies with selective *in vivo* depletion of T cells and aided with multiparametric analysis of splenocytes we demonstrate the essential role of cationic liposomes in expansion of both CD4^+^ and CD8^+^ T cells for long-lasting protection against *L. donovani* in murine model. However, since the study was carried out through intraperitoneal route of immunization, combining additional adjuvant(s) with cationic liposome would be essential to be effective through human administrable subcutaneous route.

## Ethics Statement

The study with human samples was approved by the Ethical Committee on Human Subjects, CSIR-IICB and also by School of Tropical Medicine (Kolkata, India). Written informed consents were obtained from all patients and donors for blood sampling. The studies with mice were performed according to the institutional guidelines of the Committee for the Purpose of Control and Supervision on Experimental Animals (CPCSEA), Ministry of Environment and Forest, Govt. of India, and approved by the Institutional Animal Ethics Committee (147/1999/CPSCEA) of CSIR-Indian Institute of Chemical Biology.

## Author Contributions

AS, SB, and NA conceived the study, designed the experiments, analyzed the data, and wrote the manuscript. SB, AS, RC, SE, ND, MA, AB, and US performed and optimized the experiments, prepared figures, and contributed reagents. All authors critically revised and approved the manuscript.

## Conflict of Interest Statement

The authors declare that the research was conducted in the absence of any commercial or financial relationships that could be construed as a potential conflict of interest.
