# Supplementary material for: Liposomal Elongation Factor-1α Triggers Effector CD4 and CD8 T Cells for Induction of Long-Lasting Protective Immunity against Visceral Leishmaniasis
Source: Front Immunol. 2018 Jan 30;9:18. doi: 10.3389/fimmu.2018.00018 (PMC5797590; doi:10.3389/fimmu.2018.00018)
Supplement: Supplementary file 1 [file Presentation_1.PDF]

Supplementary Material

Liposomal Elongation Factor-1α Triggers Effector CD4 and CD8 T Cells for Induction of Long-Lasting Protective Immunity against Visceral Leishmaniasis

Abdus Sabur,* Sudipta Bhowmick,* Rudra Chhajer, Sarfaraz Ahmad Ejazi, Nicky Didwania, Mohammad Asad, Anirban Bhattacharyya, Utsa Sinha, and Nahid Ali

*****Both the authors contributed equally

"Corresponding Author's Email: (nali@iicb.res.in, nahidali28@yahoo.in)"

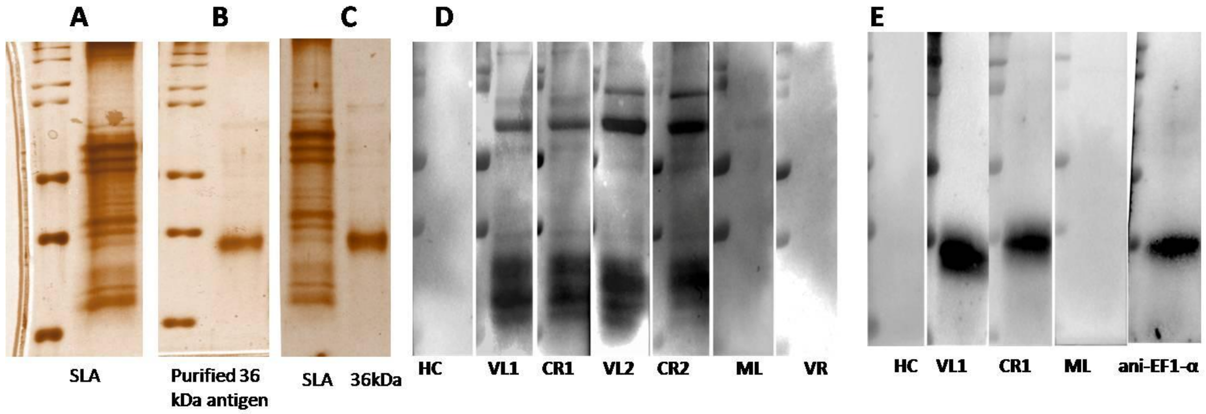

Figure S1. SDS-PAGE and western blot images of SLA and 36 kDa antigen.

Silver stained SDS-PAGE of SLA (A), 36 kDa antigen of SLA, (B), SLA and purified antigen together (C). Immunoblot of SLA with human sera (D) from Healthy (HC), VL subject 1 before (VL1) treatment and after treatment(CR1), VL subject2 before (VL2) and after treatment (CR2), Malaria (ML) and viral fever (VR). Immunoblot of 36 kDa antigen purified from SLA with sera from Healthy, VL subject 1 before (VL1) treatment and after treatment (CR1), Malaria (ML).

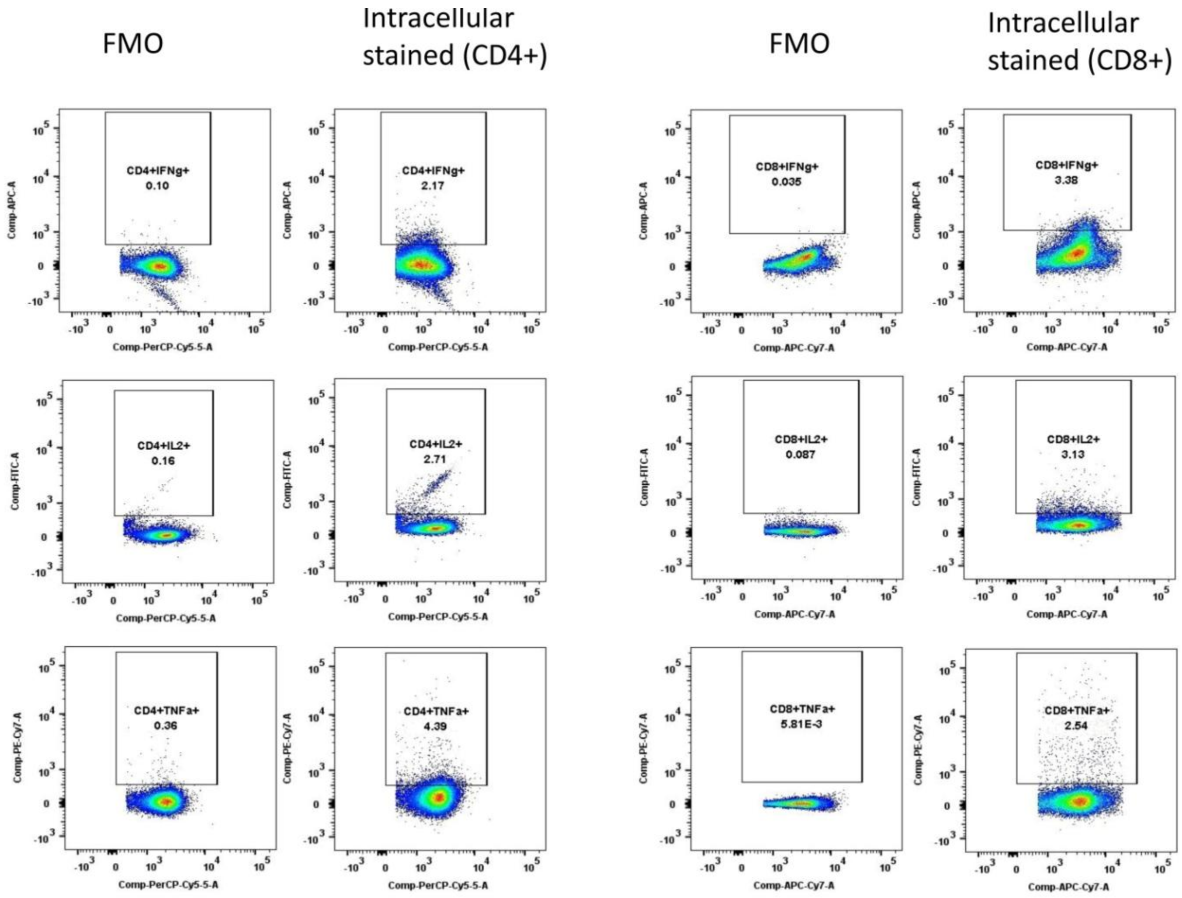

Figure S2. FMO control and intracellular cytokine gating strategy

FMO control splenoctyes were stained with surface antibodies but not intracellular cytokines. The plots are representative cytokine producing T cells in comparison with unstained FMO controls.

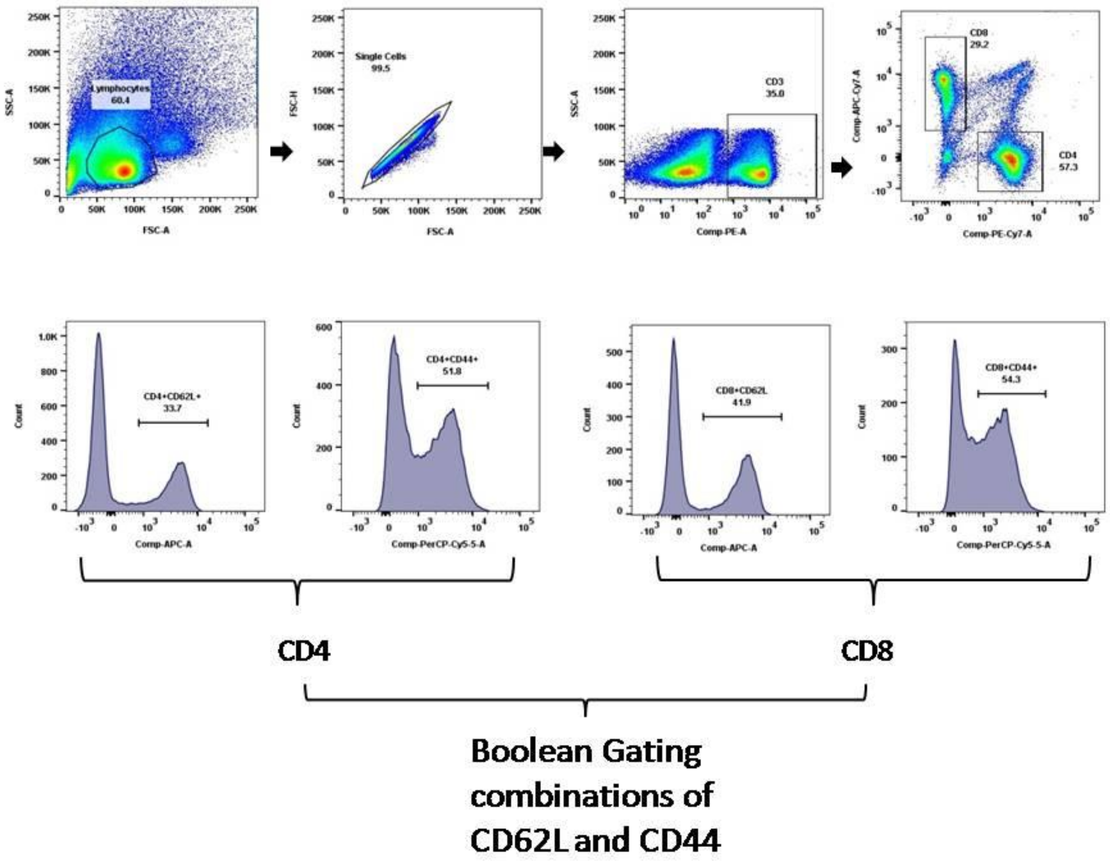

Figure S3. Representative Gating Strategy of CD62L and CD44 positive T cells.

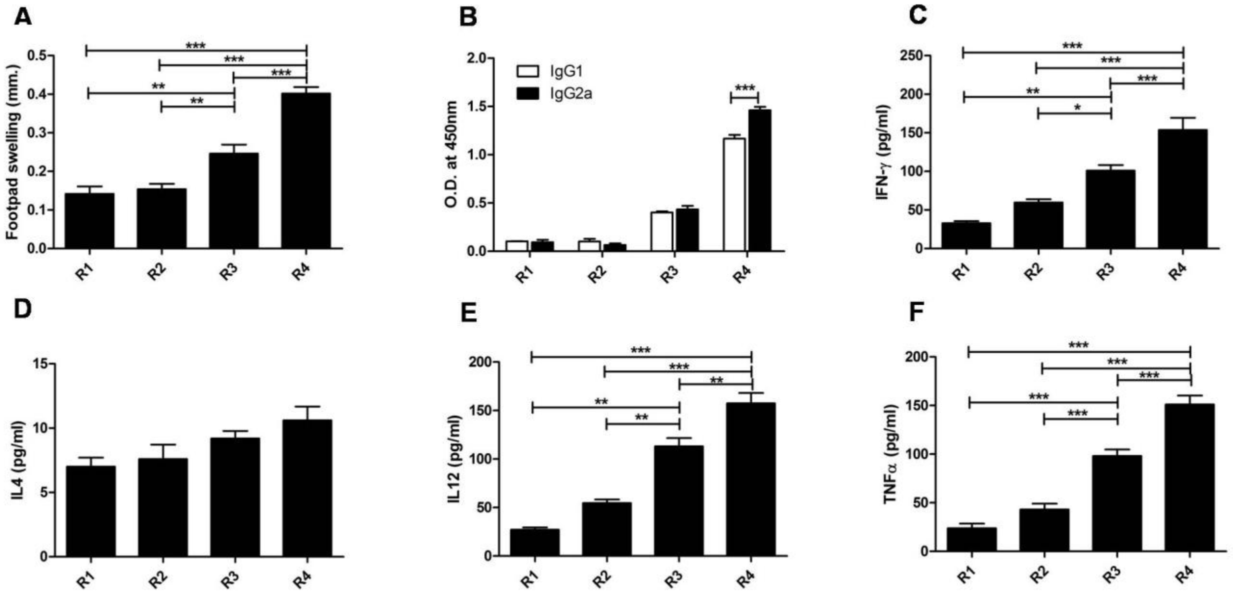

Figure S4. rEF1-α specific immune responses in BALB/c mice post immunization

Mice were immunized three times at 2-week intervals with rEF1-α alone or entrapped in cationic liposomes. Control groups received PBS or empty liposomes (Lip). Prechallenge antibody, DTH, proliferative and cytokine responses were evaluated. (*A*)DTH responses were measured as the difference (in millimeters) between the thickness of the test (LAg-injected) and control (PBS-injected) footpads at 24 h. (*B*) Ten days after the last immunization serum samples were collected and assayed for antigen-specific IgG1 and IgG2a antibodies at a dilution of 200 by indirect ELISA. Each sample was examined in duplicate. The results are shown as the mean absorbance values ± SE of five individual mice per group, representative of two independent experiments with similar results. Spleens were collected and splenocytes were stimulated in vitro with rEF1-α (5μg/ml) for 72 h. Culture supernatants were also assayed for IFN-γ (*C*), IL4 (*D*), IL-12 (*E*) and TNF-α (*F*) by capture ELISA. Each sample was examined in duplicate. The results are shown as the mean ± SE of five individual mice per group. *, $p < 0.05$; **, $p < 0.01$; ***, $p < 0.001$.

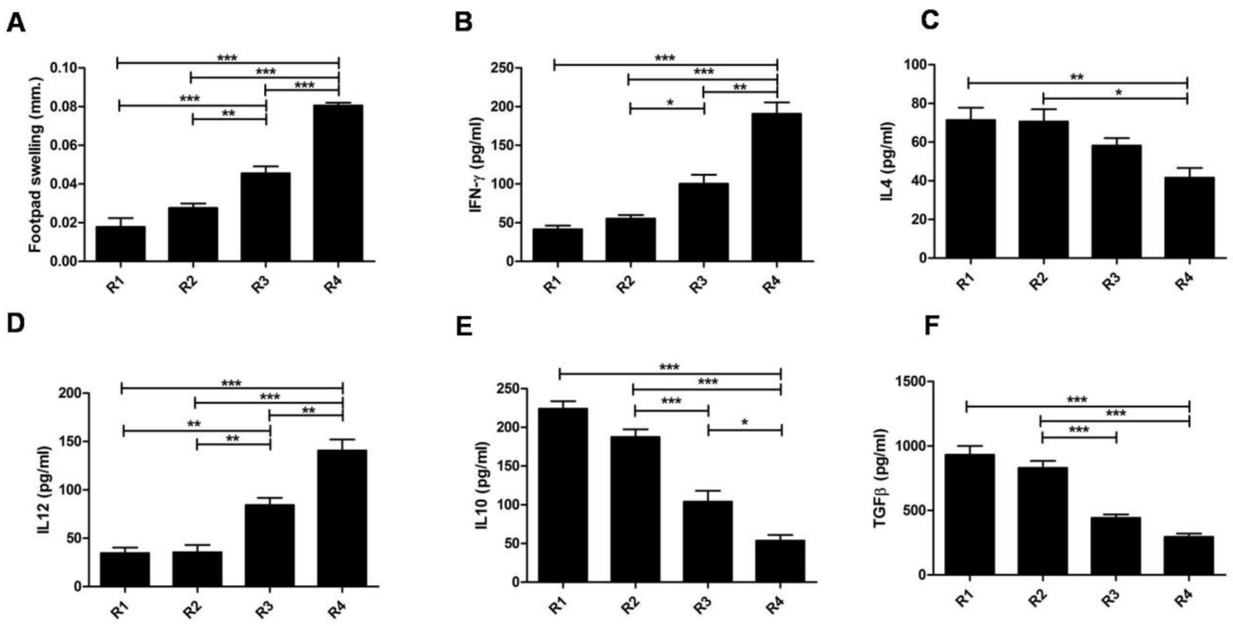

Figure S4. rEF1-α specific immune responses in immunized BALB/c mice three months post challenge infection with virulent *L. donovani*

Mice were vaccinated with rEF1-α alone or entrapped in cationic liposomes. Control groups received PBS or empty liposomes (Lip). Ten days after the last immunization all mice were challenged with $2.5X10^7$ freshly transformed *L. donovani* promastigotes through tail vain. Three months after challenge infection DTH, proliferative and cytokine responses in mice were evaluated. (*A*) DTH responses were measured as the difference (in millimeters) between the thickness of the test (LAg-injected) and control (PBS-injected) footpads at 24 h. Spleens were collected and splenocytes were stimulated in vitro with EF1-α (5μg/ml) for 72 h The culture supernatants were also assayed for IFN-γ (*B*), IL4 (*C*), IL12 (*D*), IL10 (*E*) and TGF-β (*F*) by capture ELISA. Each sample was examined in duplicate. The results are shown as the mean ± SE of five individual mice per group. *, $p < 0.05$; **, $p < 0.01$; ***, $p < 0.001$.

Table S1- Results of MALDI-TOF/TOF mass spectrometry of the 36-kDa SLA antigen

Molecular Mass (oberved)	Molecular Mass (expected)	Δ	Residues	Sequences
894.47	894.39	0.08	56-62	YAWVLDK
925.47	925.39	0.08	159-166	TVTYAQSR
975.55	975.46	0.09	236-243	LPLQDVYK
1040.45	1040.38	0.07	205-212	SDNMPWYK
1312.70	1312.59	0.11	85-96	SVFTIIDAPGHR
1762.86	1762.71	0.15	190-204	FIPISGWQGDNMIER
1778.85	1778.70	0.15	190-204	FIPISGWQGDNMIER
2542.41	2542.18	0.23	213-235	GPTLLDALDMLEPPVRPVDKPLR

Methodology for Table S1

Mass spectrometry was performed as described earlier by Bhowmick, et al 2009. Briefly, 36-kDa enriched fraction was run on SDS-PAGE, visualized with silver staining (ProteoSilver TM Plus silver stain kit, Sigma-Aldrich). The in-gel digestion was carried out according to the manufacturer's manual (Pierce). The desired band was excised and destained. The possible disulfide bonds were reduced with tris (2-carboxyethyl) phosphine (TCEP) and alkylated with iodoacetamide. Then the gel pieces were dehydrated with acetonitrile and rehydrated with 100 ng trypsin (Promega) in 25 mM ammonium bicarbonate solution and were incubated at 37°C overnight. The tryptic fragments were extracted from the gel by adding 1% trifluroacetic acid. The peptides were purified with C18 reversed-phase minicolumn filled in a micropipette tip, ZipTip C18 (Millipore). Purified peptides (0.5ml) were cocrystallized with a-cyano-4-hydroxy cinnamic acid matrix (0.5ml) (Applied Biosystems) on a matrix-assisted laser desorption ionization (MALDI) target plate. Both mass spectrometry (MS) and MS/MS spectra were acquired by matrix-assisted laser desorption ionization-time of flight (MALDI-TOF/TOF) Mass Spectrometer (Applied Biosystems 4800 Proteomics Analyzer). All spectra were collected in the reflector mode. Calibration was updated before each acquisition using a standard peptide mixture according to instrument protocol. Database searching for protein identification was performed with mass spectrometry data using GPS Explorer (Applied Biosystems) software with MASCOT (Matrix Science) search engine.
